# GJA1 rs2071165 A > G Variant Increased Gastric Cancer Risk in Females of Northwest China: A Case-Control Study

**DOI:** 10.1155/2021/5556303

**Published:** 2021-05-19

**Authors:** Lijuan Yuan, Ping Yang, Gang Wei, Jianguo Lu, Zhengyu Yang, Lin Yang, Shujia Peng, Xianli He, Guoqiang Bao

**Affiliations:** Department of General Surgery, Tangdu Hospital, The Air Force Military Medical University, Xi'an 710032, China

## Abstract

Gastric cancer (GC) is one of the most common malignancies, and its incidence rates vary widely between men and women. Previous studies have suggested that connexin 43 (Cx43, encoded by gap junction protein alpha 1 (GJA1)) and secretory carrier membrane protein 1 (SCAMP1) are key functional proteins in tumors. Herein, the association between GJA1 and SCAMP1 polymorphisms and GC susceptibility and prognosis was evaluated. A total of three single-nucleotide polymorphisms among 681GC patients and 756 controls were tested using the Agena MassARRAY RS1000 system, including GJA1 rs2071165, SCAMP1 rs4530741, and SCAMP1 rs6874309. The strength of the association with GC risk was assessed by the odds ratios (ORs) and 95% confidence intervals (CIs) generated from the logistic regression model. Kaplan–Meier curve, long-rank tests, and a multivariate Cox proportional hazard model were used for prognosis analysis. The expression of GJA1 was assessed by immunohistochemistry. The GJA1 rs2071165 AA/AG genotype significantly increased the risk of GC in the female Chinese population (OR = 1.55, 95% CI = 1.03–2.32, *p*=0.034). Furthermore, the risk effect of GJA1 rs2071165 was more evident in the subgroups of female patients with GC, stratified by age, clinical stage, tumor size, and recurrence/metastasis. However, no obvious differences in Cx43 expression in GC tissues were observed between males and females. Furthermore, no significant association between SCAMP1 rs4530741 and rs6874309 polymorphisms and GC risk or prognosis was observed. In conclusion, this study suggests for the first time that the GJA1 rs2071165 polymorphism is associated with increased GC risk in females, revealing a potential new clinical marker for assessing GC risk in females.

## 1. Introduction

Gastric cancer (GC) ranks fifth in incidence rate of all cancers and is the fourth leading cause of cancer-related deaths among all human cancers in both sexes worldwide [1].Over 1,000,000 new cases of GC and an estimated 783,000 deaths occurred globally in 2018 [2]. The highest GC incidence and mortality rates were found in East Asia [3], ranking second (13.5%) for males and fifth (7.1%) for females among the most commonly diagnosed cancers in Chinese people in 2018 [4]. GC is a multifactorial disease resulting from both environmental and genetic factors. Previous studies have shown that genetic factors, lifestyle conditions, and environmental factors play important roles in the development of GC [5].

GC incidence rates vary widely between men and women, and females show a lower overall incidence of GC compared to males [6]. Female patients also show a significantly poorer prognosis than male patients, especially among those with advanced GC aged ≤45 years [7].The reasons for such differences are not clear; however, physiological differences may be contributing factors. For example, estrogens may protect females against the development of GC [8–10]. Environmental or occupational exposures may also play a role, but the effect of smoking remains elusive [11]. In addition, although hundreds of case-control studies have examined candidate polymorphisms in relation to GC, there is still insufficient evidence for genetic differences contributing to the different incidence rates of GC between males and females.

Gap junction protein alpha 1 (GJA1), which encodes the connexin 43 (Cx43) protein, belongs to the connexin gene family and is involved in the formation of gap junction transmembrane channels, allowing the transfer of small molecules between the cytoplasm of two adjacent cells [12]. There is compelling evidence supporting the correlation between aberrant Cx43 expression and tumor growth or metastasis [13].Secretory carrier membrane protein 1 (SCAMP1) has been reported as a key functional protein in various tumors [14, 15]; it also functions as a long noncoding RNA (lncRNA) in human tumors [16, 17]. However, no studies have shown an association between polymorphisms in GJA1 and SCAMP1 and the incidence of GC. Therefore, three single-nucleotide polymorphisms (SNPs) were selected in this study, rs2071165 in GJA1, and rs4530741 and rs6874309in SCAMP1, to explore their relationship with GC risk and prognosis.

## 2. Materials and Methods

### 2.1. Study Population

In all, 1437 Han Chinese subjects were enrolled in this study, 681 of which were patients with GC that underwent radical surgery at Tangdu and Xijing Hospitals. A total of 756 healthy individuals were randomly selected through health screening at Tangdu Hospital. There were no age, sex, or disease stage restrictions for recruitment. All GC patients were unrelated, of Han Chinese descent, and newly diagnosed and histologically confirmed to have GC. Follow-up of all patients was carried out according to our standard protocol (every 6 months during the first 2 years, then once in 12 months through telephone, outpatient review, or medical records). The latest follow-up data in this analysis were obtained in October 2014. Recurrence and mortality events were recorded, and relapse-free survival (RFS) was calculated for prognosis assessment. Written permission was obtained from all participants, and the study was approved by the Institutional Review Board of the Air Force Military Medical University (Xi'an, China). The procedures were performed according to the approved guidelines and the 1964 Helsinki Declaration and its later amendments or comparable ethical standards.

### 2.2. Genotyping

To evaluate the association between the three SNPs and GC, peripheral venous blood samples (5 mL) were collected from all subjects in EDTA vacutainers. Genomic DNA was obtained from the peripheral blood lymphocytes of study subjects using the Genomic DNA Extraction Kit (Omega Bio-Tek, Norcross, GA, USA, or GoldMag Ltd., Xi'an, China) according to the manufacturers' protocol. All samples were collected before curative resection and stored at −80°C for subsequent analysis. The GJA1 gene rs2071165 G > A, SCAMP1 gene rs4530741 A > C, and SCAMP1 gene rs6874309 T > A polymorphisms were genotyped on the Agena MassARRAY RS1000 platform according to the standard protocol (Applied Biosystems, Foster City, CA, USA). Primers were designed using the Agena MassARRAY Assay Design 4.0 software.

### 2.3. Statistical Analysis

Analyses were performed using SPSS version 20.0. Student's *t*-test was used to compare differences in age between the two groups. The chi-square or Fisher's exact test was used for sex and genotype frequency estimation. The odds ratio (OR) and confidence interval (CI) values of associations of genotype frequencies were calculated using binary logistic regression with the SNPStats web tool (http://bioinfo.iconcologia.net/snpstats/start.htm), adjusting for age and gender. Kaplan–Meier curves and log-rank tests were also used to estimate associations between SNPs and overall survival (OS) and RFS. The Cox proportional hazard regression model was applied to calculate hazard ratios (HRs) and 95% CIs for predicting the effects of the SNPs on GC prognosis. All statistical analyses were two-sided, and *p* < 0.05 was considered statistically significant.

### 2.4. Immunohistochemistry

GC tissue specimens were collected from 45 patients (33 males and 12 females), and 33 nontumor adjacent normal tissue samples were obtained from a segment of the resected specimens that was furthest from the tumor (>5 cm) (23 males and 10 females). All patients were pathologically diagnosed postoperatively. Written informed consent was obtained from all participants.

Immunohistochemical (IHC) analysis was performed on paraffin-embedded tissue specimens. The slides were incubated in 0.3% H_2_O_2_ in methanol for 20 min. For antigen retrieval, the slides were boiled in 10-mM sodium citrate buffer (pH 6.0) in a microwave oven for 15 min. After blocking nonspecific binding with 5% BSA for 1 h, the slides were incubated with an anti-connexin 43 antibody (1 : 100, Abcam, Cambridge) overnight at 4°C. The slides were incubated with a biotinylated sheep anti-rabbit secondary antibody and 3,3ʹ-diaminobenzidine (DAB). The specific immunoreactivity showed clear brown staining.

Semiquantitative counts of the staining were scored according to Barne's method. Assessment of the score standard was based on staining intensity and percentage of positive cells. Immunostaining results were scored as the sum of the extent and intensity of immunoreactivity.

All data were analyzed using the GraphPad Prism 8.0 software. A *t*-test was used for comparison between groups. A *p* value <0.05 was considered statistically significant.

## 3. Results

The genotype frequencies and their associations with the risk of GC in the Han Chinese population are shown in Table 1. Three genotypes were detected at each single-nucleotide polymorphism (SNP) locus with similar frequencies of each genotype in the case and control groups, respectively. The *p* value of each SNP from the Hardy–Weinberg equilibrium (HWE) test was >0.05. Moreover, no significant association was observed between each SNP and GC susceptibility without regard to gender differences.

Furthermore, stratification analysis was performed to evaluate the association between the polymorphisms and GC risk (Table 2). A significant association between GJA1 polymorphisms and GC risk in females was observed. Compared to the GJA1 rs2071165 GG genotype, the dominant model demonstrated that the combined genotype AG/AA was significantly associated with an increased risk of GC in women, after adjusting for age (OR = 1.55, 95% CI = 1.03–2.32, *p*=0.034). The frequencies of AA and AG genotypes in female patients were higher compared with those in female controls, whereas the frequencies of GG genotypes in female cases were lower compared with those in female controls (Supplementary Table 2). No significant associations were observed in men. In addition, no significant association between the AG/AA genotypes and other subgroups was observed, stratifying by age, clinical stages, tumor size, tumor position, and recurrence/metastasis. Moreover, there were no significant differences in specific genotypes or allelic frequencies associated with the prognosis of GC (Table 3 and Supplementary Table 5).

Further stratified analyses based on various female patient characteristics were performed. As shown in Table 4 (Supplementary Table 4), after adjusting for age, the dominant model demonstrated that GJA1 rs2071165 combined genotype AA/AG was significantly associated with an increased risk of GC in female subjects aged <55 years (OR = 2.06, 95% CI = 1.01–4.21, *p*=0.046), when compared to the rs2071165 GG genotype. Moreover, the AA/AG genotype was associated with an increased risk of GC for females with a tumor size ≥5 cm (OR = 1.75, 95% CI = 1.03–2.99, *p*=0.04), females in tumor stage III/IV (OR = 2.09, 95% CI = 1.12–3.91, *p*=0.02), and females showing negative recurrence/metastasis (OR = 1.93, 95% CI = 1.16–3.23, *p*=0.01), compared to the rs2071165 GG genotype. Furthermore, the codominant model showed that the GJA1 rs2071165 AA genotype had a significant association with an increased risk of GC in women with tumor stage III/IV (OR = 4.19, 95% CI = 1.41–12.45, *p*=0.01) and the rs2071165 AA and AG genotypes were significantly associated with an increased risk of GC in women with negative recurrence/metastasis (OR = 2.84, 95% CI = 1.03–7.80, *p*=0.03; OR = 1.82, 95% CI = 1.06–3.10, *p*=0.03, respectively). The recessive model also showed that the GJA1 rs2071165 AA genotype was associated with an increased risk of GC in females with stage III/IV tumors compared to the rs2071165 AG/GG genotype (OR = 3.21, 95% CI = 1.14–9.08, *p*=0.03).

**Figure 1:**
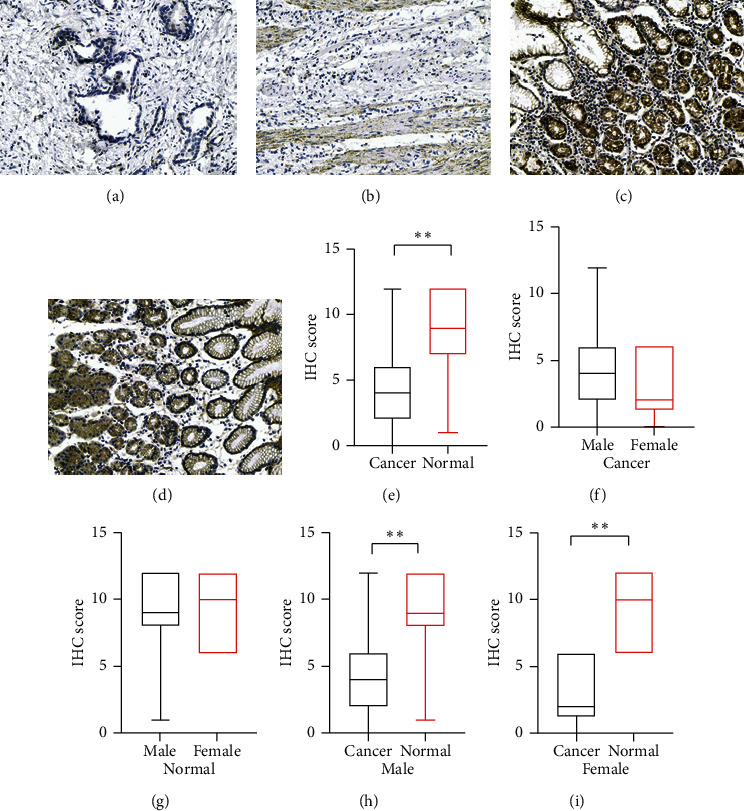
Connexin 43 (Cx43) expression in normal gastric tissue and gastric cancer tissue specimens. (a, b) Cx43 expression in gastric cancer tissues; (c, d) Cx43 expression in normal gastric tissues; (e–i) immunohistochemistry (IHC) results for Cx43 expression in gastric cancer tissues and normal gastric tissues. *∗∗p* < 0.01.

To investigate differences in Cx43 expression in gastric tissue between males and females, the expression of Cx43 in GC and adjacent normal gastric tissue was assessed in a different cohort with GC, as the resected tissue sample of the original study population of GC patients was not retained. Decreased expression of Cx43 in GC was observed, but no significant difference in Cx43 expression was observed between males and females (Figure 1).

## 4. Discussion

The associations of GJA1 rs2071165 and SCAMP1 rs4530741 A > C and rs6874309 T > A polymorphisms with GC risk and prognosis were investigated in this study. The GJA1 gene rs2071165 AA/AG genotype significantly increased the risk of GC in the female Chinese population, which indicated that GJA1polymorphisms may contribute to GC susceptibility in females. Furthermore, the risk effect of GJA1 rs2071165 polymorphisms was more evident in the subgroups of female patients with GC, stratified by age, clinical stage, tumor size, and recurrence/metastasis. Negative results were observed for SCAMP1 rs4530741 A > C and rs6874309 T > A polymorphisms. To the best of our knowledge, this is the first report documenting an association between GJA1and GC risk. Once validated, GJA1 may be used as a new marker for assessing GC risk in females, combined with traditional clinical risk factors.

Connexin 43 is a member of the connexin family and known for its greater capacity for transporting macromolecules than other connexin proteins [18].Compelling evidence suggests that dysregulated Cx43 (GJA1) expression is associated with tumor development and progression [19–21], making Cx43 an attractive tumor biomarker. However, its role in cancer progression and metastasis remains controversial [13]. Decreased expression of Cx43 was found in primary GC, while increased Cx43 expression was found to contribute to lymph node metastasis [22]. Increased Cx43 expression has been reported to be associated with poor prognosis in some cancer types [13, 19, 23], whereas the contrary has been reported in breast cancer [24]. However, the current consensus appears to be that the loss of Cx43 gap junction intercellular communication is an early event in malignancy, with the possibility of gap junction restoration in the event of metastasis [25], which also enhances the role of Cx43 in cancer development and prognosis.

Despite extensive investigations of Cx43(GJA1) expression and its corresponding activity in cancer evolution, few studies have focused on the effect of SNPs in GJA1 on cancer risk or prognosis. According to web-based SNP selection tools (https://manticore.niehs.nih.gov/snpinfo/snpfunc.html), two functional SNPs were selected in the GJA1 rs2071165 gene region for further analysis in our study.rs2071165is located in the upstream-variant-2KB region ofGJA1 and is predicted to be a transcription factor binding site, which may influence the expression of Cx43 in GC patients. The correlation between rs2071165 and cancer risk has not yet been investigated. The SNP rs2071166 was removed from this study due to its strong linkage disequilibrium with the SNP rs2071165. However, the AA/AG genotypes of functional SNP rs2071165 were significantly associated with GC risk in females and the variant-containing (AA, AG, and AA/AG) genotypes showed a more prominent effect on subgroups of female GC patients, stratifying by age, clinical stages, tumor size, and recurrence/metastasis, supporting the important role of Cx43 in GC development. However, no significant association between rs2071165 polymorphisms and GC prognosis was observed in this study, even in females.

Sex disparity in GC has been proven [7]. In the present study, it was observed that the GJA1 rs2071165 AA/AG genotype was significantly associated with an increased risk of GC in females but not in males. Cx43 is hormone-responsive [26], and the inhibition of estrogen receptors could reduce connexin 43 expression in breast cancers [27]. Estrogen also has a preventive role in GC [28]. Furthermore, a report suggested that hypothalamic Cx43 expression is regulated by steroid hormones in a brain-region-specific and sexually dimorphic manner [29]. The interaction between estrogen and aberrant Cx43 expression might also contribute to GC development and progression. However, no significant difference in Cx43 expression was observed between males and females in this study. This finding may be limited by the small sample size. Therefore, more evidence and sample validation is needed to support this hypothesis.

Our study has several limitations. First, the exact mechanism of GJA1 polymorphism needs to be further clarified, even though a correlation between GJA1 rs2071165 polymorphisms and GC risk was observed. Second, the sample size was too small to have enough statistical power for the stratified analyses in females. Only two SNPs in SCAMP1 were evaluated; other important SNPs may have been neglected. Third, the association between genetic and environmental factors, such as dietary habits or the presence of *H. pylori* infection, was not considered here due to the lack of these data. Moreover, the study was restricted to the Han Chinese population; therefore, generalizability issues cannot be ruled out. Further studies on larger populations, including other ethnicities, are warranted.

## 5. Conclusions

The study suggests that GJA1 rs2071165 polymorphisms are associated with increased GC risk in females, but no significant association between SCAMP1 rs4530741 and rs6874309 polymorphisms and GC risk or prognosis was observed. Moreover, the GJA1 rs2071165 polymorphisms may contribute to an increased risk of GC in women aged <55 years. The present study shows the potential clinical significance of GJA1 rs2071165 polymorphisms in predicting GC in women and a hypothesis for the sex difference in incidence of GC.

## Figures and Tables

**Table 1:** Genetic variants associated with the susceptibility of gastric cancer.

Model	Genotype	Control	Case	OR^2^ (95% CI)	*p*	OR (95% CI)	*p* ^1^
rs2071165	HWE^3^*p*=0.699						
Codominant	GG	423	382	1.00	0.99	1.00	0.96
	AG	288	257	0.98 (0.79–1.22)		1.03 (0.82–1.30)	
	AA	45	41	1.01 (0.64–1.57)		1.03 (0.65–1.64)	

Dominant	GG	423	382	1.00	0.89	1.00	0.77
	AG-AA	333	298	0.99 (0.80–1.21)		1.03 (0.83–1.29)	

**rs4530741**	HWE *p*=0.121						
Codominant	CC	250	228	1.00	0.43	1.00	0.33
	AC	351	330	1.03 (0.82–1.30)		1.04 (0.81–1.32)	
	AA	156	122	0.86 (0.64–1.15)		0.83 (0.61–1.14)	

Dominant	CC	250	228	1.00	0.84	1.00	0.81
	AC-AA	507	452	0.98 (0.78–1.22)		0.97 (0.77–1.22)	

rs6874309	HWE *p*=0.246						
Codominant	AA	635	571	1.00	0.6	1.00	0.78
	AT	114	106	1.03 (0.78–1.38)		0.96 (0.71–1.30)	
	TT	8	4	0.56 (0.17–1.86)		0.65 (0.18–2.37)	

Dominant	AA	635	571	1.00	0.98	1.00	0.7
	AT-TT	122	110	1.00 (0.76–1.33)		0.94 (0.70–1.27)	

^1^Adjust by age and gender. ^2^Odds ratio. ^3^Hardy–Weinberg equilibrium.

**Table 2:** Stratification analyses for the association between genetic polymorphism and gastric cancer susceptibility.

	Genotype	Control	Case	OR (95% CI)	*p*	OR (95% CI)	*p* ^2^
Age^1^							
<55	rs2071165						
	GG	235	133	1.00	0.36	1.00	0.32
	AG	173	98	1.00 (0.72–1.38)		1.02 (0.73–1.42)	
	AA	21	19	1.60 (0.83–3.08)		1.66 (0.86–3.22)	
	AG/AA	194	117	1.06 (0.78–1.45)	0.71	1.09 (0.80–1.49)	0.59
	**rs4530741**						
	CC	127	84	1.00	0.37	1.00	0.46
	AC	213	122	0.87 (0.61–1.23)		0.89 (0.62–1.27)	
	AA	90	43	0.72 (0.46–1.14)		0.75 (0.47–1.18)	
	AC/AA	303	165	0.82 (0.59–1.15)	0.26	0.84 (0.60–1.18)	0.33
	**rs6874309**						
	AA	358	215	1.00	0.63	1.00	0.68
	AT	68	33	0.81 (0.52–1.27)		0.83 (0.53–1.30)	
	TT	4	2	0.83 (0.15–4.58)		0.76 (0.14–4.27)	
	AT/TT	72	35	0.81 (0.52–1.25)	0.34	0.82 (0.53–1.28)	0.39

≥55	rs2071165						
	GG	188	249	1.00	0.44	1.00	0.36
	AG	115	158	1.04 (0.76–1.41)		1.15 (0.81–1.63)	
	AA	24	22	0.69 (0.38–1.27)		0.70 (0.35–1.39)	
	AG/AA	139	180	0.98 (0.73–1.31)	0.88	1.07 (0.77–1.49)	0.69
	**rs4530741**						
	CC	123	142	1.00	0.22	1.00	0.7
	AC	138	208	1.31 (0.94–1.80)		1.15 (0.80–1.66)	
	AA	66	79	1.04 (0.69–1.56)		1.00 (0.63–1.59)	
	AC/AA	204	287	1.22 (0.90–1.65)	0.2	1.11 (0.79–1.55)	0.56
	**rs6874309**						
	AA	277	354	1.00	0.29	1.00	0.48
	AT	46	73	1.24 (0.83–1.85)		1.18 (0.75–1.85)	
	TT	4	2	0.39 (0.07–2.15)		0.38 (0.05–3.02)	
	AT/TT	50	75	1.17 (0.79–1.74)	0.42	1.13 (0.72–1.75)	0.6

Gender^3^							
Male	rs2071165						
	GG	262	305	1.00	0.35	1.00	0.74
	AG	194	191	0.85 (0.65–1.10)		0.92 (0.69–1.22)	
	AA	33	30	0.78 (0.46–1.32)		0.84 (0.47–1.48)	
	AG/AA	227	221	0.84 (0.65–1.07)	0.16	0.91 (0.69–1.19)	0.48
	**rs4530741**						
	CC	150	167	1.00	0.77	1.00	0.65
	AC	242	263	0.98 (0.74–1.29)		1.04 (0.76–1.41)	
	AA	97	95	0.88 (0.61–1.26)		0.87 (0.59–1.29)	
	AC/AA	339	358	0.95 (0.73–1.24)	0.70	0.99 (0.74–1.32)	0.93
	**rs6874309**						
	AA	406	441	1.00	0.45	1.00	0.43
	AT	78	83	0.98 (0.70–1.37)		0.89 (0.62–1.29)	
	TT	5	2	0.37 (0.07–1.91)		0.33 (0.05–2.24)	
	AT/TT	83	85	0.94 (0.68–1.31)	0.73	0.86 (0.60–1.24)	0.43

Female	rs2071165						
	GG	161	77	1.00	0.1	1.00	0.084
	AG	94	66	1.47 (0.97–2.22)		1.49 (0.98–2.26)	
	AA	12	11	1.92 (0.81–4.54)		2.04 (0.85–4.87)	
	AG/AA	106	77	**1.52 (1.02–2.27)**	**0.04**	**1.55 (1.03–2.32)**	**0.034**
	rs4530741						
	CC	100	60	1.00	0.52	1.00	0.65
	AC	108	67	1.03 (0.66–1.61)		1.00 (0.64–1.57)	
	AA	59	27	0.76 (0.44–1.33)		0.79 (0.45–1.38)	
	AC/AA	167	94	0.94 (0.62–1.41)	0.76	0.93 (0.61–1.40)	0.72
	rs6874309						
	AA	228	129	1.00	0.9	1.00	0.84
	AT	36	23	1.13 (0.64–1.99)		1.19 (0.67–2.11)	
	TT	3	2	1.18 (0.19–7.14)		1.08 (0.17–6.69)	
	AT/TT	39	25	1.13 (0.66–1.96)	0.66	1.18 (0.68–2.05)	0.56

Recurrence and metastasis^a^							
Negative	rs2071165						
	GG	423	208	1.00	0.86	1.00	0.76
	AG	288	148	1.05 (0.81–1.35)		1.08 (0.83–1.42)	
	AA	45	20	0.90 (0.52–1.57)		0.90 (0.50–1.61)	
	AG/AA	333	168	1.03 (0.80–1.32)	0.84	1.06 (0.81–1.38)	0.67
	**rs4530741**						
	CC	124	250	1.00	0.63	1.00	0.61
	AC	181	250	1.04 (0.79–1.38)		1.00 (0.75–1.35)	
	AA	71	156	0.92 (0.64–1.31)		0.9075 (0.62–1.32)	
	AC/AA	252	506	1.00 (0.77–1.31)	0.98	0.9745 (0.74–1.29)	0.86
	**rs6874309**						
	AA	310	634	1.00	0.42	1.00	0.72
	AT	64	114	1.15 (0.82–1.61)		1.07 (0.75–1.52)	
	TT	2	8	0.51 (0.11–2.42)		0.7212 (0.14–3.97)	
	AT/TT	66	122	1.11 (0.80–1.54)	0.55	1.05 (0.74–1.49)	0.79

Positive	rs2071165						
	GG	423	167	1.00	0.75	1.00	0.97
	AG	288	102	0.90 (0.67–1.20)		0.96 (0.71–1.30)	
	AA	45	18	1.01 (0.57–1.80)		0.99 (0.54–1.80)	
	AG/AA	333	120	0.91 (0.69–1.20)	0.51	0.96 (0.72–1.28)	0.8
	**rs4530741**						
	CC	99	250	1.00	0.98	1.00	0.72
	AC	138	350	0.99 (0.73–1.35)		1.06 (0.77–1.46)	
	AA	49	156	0.80 (0.538–1.18)		0.80 (0.53–1.21)	
	AC/AA	187	506	0.93 (0.70–1.24)	0.64	0.98 (0.73–1.32)	0.88
	**rs6874309**						
	AA	245	634	1.00	0.63	1.00	0.41
	AT	40	114	0.91 (0.62–1.34)		0.84 (0.57–1.26)	
	TT	2	8	0.65 (0.14–3.07)		0.62 (0.12–3.21)	
	AT/TT	42	122	0.89 (0.61–1.30)	0.55	0.83 (0.56–1.23)	0.36

Clinical stage^a^							
**Early**	rs2071165						
	GG	71	423	1.00	0.22	1.00	0.10
	AG	61	288	1.26 (0.87–1.83)		1.39 (0.94–2.05)	
	AA	9	45	1.19 (0.56–2.54)		1.17 (0.53–2.57)	
	AG/AA	70	333	1.25 (0.87–1.80)	0.22	1.35 (0.93–1.87)	0.12
	**rs4530741**						
	CC	38	250	1.00	0.13	1.00	0.12
	AC	74	350	1.39 (0.91–2.12)		1.42 (0.91–2.21)	
	AA	28	156	1.18 (0.70–2.00)		1.23 (0.71–2.13)	
	AC/AA	102	506	1.33 (0.89–1.98)	0.17	1.36 (0.90–2.07)	0.15
	**rs6874309**						
	AA	112	634	1.00	0.16	1.00	0.32
	AT	28	114	1.39 (0.88–2.20)		1.27 (0.79–2.06)	
	TT	0	8	NA		NA	
	AT/TT	28	122	1.30 (0.82–2.052)	0.26	1.2 (0.74–1.94)	0.45

**Middle**	rs2071165						
	GG	272	423	1.00	0.43	1.00	0.62
	AG	168	288	0.91 (0.71–1.16)		0.94 (0.73–1.21)	
	AA	25	45	0.86 (0.52–1.44)		0.85 (0.50–1.45)	
	AG/AA	193	333	0.90 (0.71–1.14)	0.38	0.93 (0.73–1.18)	0.53
	**rs4530741**						
	CC	166	250	1.00	0.60	1.00	0.67
	AC	217	350	0.93 (0.72–1.21)		0.94 (0.72–1.24)	
	AA	82	156	0.79 (0.57–1.10)		0.77 (0.55–1.09)	
	AC/AA	299	506	0.89 (0.70–1.13)	0.35	0.89 (0.69–1.15)	0.37
	**rs6874309**						
	AA	395	634	1.00	0.73	1.00	0.49
	AT	67	114	0.94 (0.68–1.31)		0.89 (0.63–1.25)	
	TT	4	8	0.80 (0.24–2.68)		0.93 (0.26–3.38)	
	AT/TT	71	122	0.93 (0.68–1.28)	0.67	0.89 (0.64–1.24)	0.49

**Late**	rs2071165						
	GG	32	423	1.00	0.77	1.00	0.98
	AG	20	288	0.92 (0.51–1.64)		1.01 (0.55–1.83)	
	AA	3	45	0.88 (0.26–2.99)		0.76 (0.21–2.67)	
	AG/AA	23	333	0.91 (0.52–1.59)	0.75	0.97 (0.54–1.71)	0.90
	**rs4530741**						
	CC	19	250	1.00	0.96	1.00	0.66
	AC	27	350	1.02 (0.55–1.87)		1.15 (0.61–2.18)	
	AA	9	156	0.76 (0.34–1.72)		0.90 (0.39–2.10)	
	AC/AA	36	506	0.94 (0.53–1.67)	0.82	1.08 (0.59–1.97)	0.81
	**rs6874309**						
	AA	46	634	1.00	0.82	1.00	0.88
	AT	9	114	1.09 (0.52–2.28)		0.94 (0.44–2.03)	
	TT	0	8	NA		NA	
	AT/TT	9	122	1.02 (0.49–2.13)	0.96	0.89 (0.41–1.91)	0.75

Tumor size^a^							
≥5 cm	rs2071165			1.00		1.00	
	GG	423	145	1.00	0.44	1.00	0.44
	AG	288	106	1.07 (0.80–1.44)		1.14 (0.84–1.55)	
	AA	45	22	1.43 (0.83–2.46)		1.40 (0.79–2.48)	
	AG/AA	333	128	1.12 (0.85–1.48)	0.42	1.18 (0.88–1.58)	0.27
	**rs4530741**						
	CC	93	250	1.00	0.86	1.00	0.69
	AC	134	350	1.03 (0.75–1.40)		1.07 (0.77–1.48)	
	AA	45	156	0.78 (0.52–1.17)		0.81 (0.53–1.24)	
	AC/AA	179	506	0.95 (0.71–1.27)	0.74	0.99 (0.73–1.34)	0.94
	**rs6874309**						
	AA	239	634	1.00	0.21	1.00	0.15
	AT	33	114	0.77 (0.51–1.16)		0.73 (0.47–1.12)	
	TT	1	8	0.33 (0.04–2.67)		0.37 (0.04–3.31)	
	AT/TT	34	122	0.74 (0.49–1.11)	0.15	0.71 (0.46–1.08)	0.11

<5 cm	rs2071165						
	GG	423	224	1.00	0.28	1.00	0.36
	AG	288	140	0.92 (0.71–1.19)		0.97 (0.74–1.27)	
	AA	45	15	0.63 (0.34–1.15)		0.64 (0.34–1.20)	
	AG/AA	333	155	0.88 (0.68–1.13)	0.31	0.92 (0.71–1.20)	0.55
	**rs4530741**						
	CC	126	250	1.00	0.86	1.00	0.89
	AC	181	350	1.03 (0.78–1.36)		1.02 (0.76–1.37)	
	AA	72	156	0.92 (0.64–1.30)		0.89 (0.63–1.29)	
	AC/AA	253	506	0.99 (0.76–1.29)	0.95	0.98 (0.75–1.29)	0.89
	**rs6874309**						
	AA	306	634	1.00	0.15	1.00	0.38
	AT	70	114	1.27 (0.92–1.77)		1.16 (0.83–1.64)	
	TT	3	8	0.78 (0.20–2.95)		0.88 (0.21–3.66)	
	AT/TT	73	122	1.24 (0.90–1.71)	0.19	1.15 (0.82–1.61)	0.42

Position^a^							
Cardia	rs2071165						
	GG	423	78	1.00	0.14	1.00	0.34
	AG	288	39	0.73 (0.49–1.11)		0.80 (0.51–1.27)	
	AA	45	7	0.84 (0.37–1.94)		0.75 (0.30–1.87)	
	AG/AA	333	46	0.75 (0.51–1.11)	0.15	0.79 (0.51–1.23)	0.30
	**rs4530741**						
	CC	38	250	1.00	0.59	1.00	0.66
	AC	60	350	1.13 (0.73–1.75)		1.12 (0.68–1.83)	
	AA	27	156	1.14 (0.67–1.94)		1.38 (0.76–2.50)	
	AC/AA	87	506	1.13 (0.75–1.71)	0.56	1.19 (0.75–1.89)	0.46
	**rs6874309**						
	AA	107	634	1.00	0.66	1.00	0.25
	AT	17	114	0.88 (0.51–1.53)		0.70 (0.38–1.30)	
	TT	1	2	0.74 (0.09–5.98)		0.83 (0.067–10.18)	
	AT/TT	18	333	0.87 (0.51–1.49)	0.62	0.70 (0.38–1.29)	0.26

Noncardia	rs2071165						
	GG	423	238	1.00	1.00	1.00	0.79
	AG	288	162	1.00 (0.78–1.28)		1.04 (0.80–1.34)	
	AA	45	25	0.99 (0.59–1.65)		0.98 (0.58–1.66)	
	AG/AA	333	187	1.00 (0.79–1.27)	0.99	1.03 (0.80–1.31)	0.83
	**rs4530741**						
	CC	142	250	1.00	0.82	1.00	0.70
	AC	205	350	1.03 (0.79–1.35)		1.06 (0.80–1.40)	
	AA	76	156	0.86 (0.61–1.21)		0.85 (0.60–1.21)	
	AC/AA	281	506	0.98 (0.76–1.26)	0.86	0.99 (0.76–1.29)	0.95
	**rs6874309**						
	AA	350	634	1.00	0.47	1.00	0.80
	AT	71	114	1.13 (0.82–1.56)		1.04 (0.75–1.46)	
	TT	3	8	0.68 (0.18–2.58)		0.75 (0.18–3.04)	
	AT/TT	74	122	1.10 (0.80–0.58)	0.56	1.03 (0.74–1.43)	0.87

^1^Adjusted by gender; ^2^Adjusted by age and gender; ^3^Adjusted by age. ^a^Patient numbers may not add up to 100% of available subjects because of missing clinical data.

**Table 3:** Association of genetic polymorphisms and gastric cancer prognosis.

	Genotype	OS^3^						RFS^4^					
		Total	Event	Log-rank *p*	MST^1^	HR^2^ (95% CI)	*p*	Total	Event	Log-rank *p*	MST^1^	Hr (95% CI)	*p*
rs2071165	GG	375	125	0.77	57	1.00		375	167	0.46	34	1.00	
	AG	250	85		62	1.03 (0.75–1.41)	0.87	250	102		39.52	0.86 (0.64–1.15)	0.30
	AA	38	15		40	1.01 (0.54–1.87)	0.98	38	18		25	1.34 (0.77–2.32)	0.30
	Dominant	288	100	0.94	62	1.02 (0.76–1.39)	0.88	288	120	0.65	51	0.91 (0.69–1.20)	0.50

rs4530741	CC	223	79	0.95	56	1.00		223	99	0.75	33	1.00	
	AC	319	110		59	1.05 (0.75–1.48)	0.76	319	138		37	0.99 (0.73–1.33)	0.93
	AA	120	36		43.12	1.03 (0.66–1.60)	0.91	120	49		25	1.09 (0.74–1.60)	0.67
	Dominant	439	146	0.80	62	1.05 (0.76–1.44)	0.78	439	187	0.58	37	1.01 (0.76–1.34)	0.93

rs6874309	AA	555	190	0.79	57	1.00		555	245	0.51	32	1.00	
	TA	104	35		42.985	1.14 (0.75–1.71)	0.55	104	40		39.79	0.92 (0.631–1.34)	0.67
	TT	4	1		55	0.56 (0.08–1.09)	0.57	4	2		50	0.90 (0.20–3.67)	0.88
	Dominant	108	36	0.86	57	1.10 (0.73–1.65)	0.65	108	42	0.24	50	0.92 (0.64–1.33)	0.66

^1^Median survival time, mean survival time was provided when MST could not be calculated; ^2^hazard ratio; ^3^overall survival; ^4^relapse free survival.

**Table 4:** Stratification analyses for the association between GJA1 rs2071165 G> A polymorphism and gastric cancer susceptibility in females.

	Model	Genotype	Case	Control	OR (95% CI)	*p*	OR (95% CI)	*p* ^1^
Age								
≥55	Codominant	GG	38	105	1.00	0.26	1.00	0.25
		AG	33	66	1.38 (0.79–2.42)		1.42 (0.78–2.56)	
		AA	6	9	1.84 (0.61–5.52)		2.15 (0.67–6.88)	
	Dominant	GG	38	105	1.00	0.19	1.00	0.16
		AA/AG	39	75	1.44 (0.84–2.46)		1.50 (0.85–2.64)	
	Recessive	AG/GG	71	171	1.00	0.39	1.00	0.28
		AA	6	9	1.61 (0.55–4.68)		1.86 (0.60–5.75)	

<55	Codominant	GG	39	56	1.00	0.11	1.00	0.10
		AG	33	28	1.69 (0.88–3.24)		1.85 (0.88–3.88)	
		AA	5	3	2.39 (0.54–10.60)		4.52 (0.93–22.00)	
	Dominant	GG	39	56	1.00	0.08	1.00	**0.046**
		AA/AG	38	31	1.76 (0.94–3.93)		**2.06 (1.01–4.21)**	
	Recessive	AG/GG	72	84	1.00	0.37	1.00	0.11
		AA	5	3	1.94 (0.45–8.42)		3.51 (0.75–16.53)	

Tumor size^a^								
≥5 cm	Codominant	GG	32	161	1.00	0.07	1.00	0.08
		AG	31	94	1.66 (0.95–2.89)		1.66 (0.95–2.89)	
		AA	6	12	2.52 (0.88–7.20)		2.53 (0.88–7.23)	
	Dominant	GG	32	161	1.00	**0.04**	1.00	**0.04**
		AA/AG	37	106	**1.76 (1.03–2.99)**		**1.753 (1.03–2.99)**	
	Recessive	AG/GG	63	255	1.00	0.17	1.00	0.17
		AA	6	12	2.02 (0.73–5.60)		2.04 (0.73–5.64)	

<5 cm	Codominant	GG	41	161	1.00	0.17	1.00	0.21
		AG	34	94	1.42 (0.84–2.39)		1.42 (0.82–2.46)	
		AA	5	12	1.64 (0.55–4.91)		2.01 (0.64–6.34)	
	Dominant	GG	41	161	1.00	0.15	1.00	0.15
		AA/AG	39	106	1.45 (0.87–2.39)		1.48 (0.87–2.51)	
	Recessive	AG/GG	75	255	1.00	0.52	1.00	0.33
		AA	5	12	1.42 (0.48–4.15)		1.75 (0.57–5.36)	

Clinical stage^a^								
0/I/II*∗*	Codominant	GG	56	161	1.00	0.26	1.00	0.19
		AG	43	94	1.32 (0.82–2.11)		1.38 (0.85–2.23)	
		AA	5	12	1.20 (0.40–3.55)		1.28 (0.42–3.85)	
	Dominant	GG	56	161	1.00	0.26	1.00	0.17
		AA/AG	48	106	1.30 (0.82–2.06)		1.37 (0.86–2.18)	
	Recessive	AG/GG	99	255	1.00	0.90	1.00	0.84
		AA	5	12	1.07 (0.37–3.13)		1.12 (0.38–3.32)	

III/IV	Codominant	GG	20	161	1.00	0.01	1.00	**0.01**
		AG	22	94	1.88 (0.98–3.63)		1.83 (0.94–3.54)	
		AA	6	12	4.03 (1.36–11.91)		4.19 (1.41–12.45)	
	Dominant	GG	20	161	1.00	0.02	1.00	**0.02**
		AA/AG	28	106	2.13 (1.14–3.97)		**2.09 (1.12–3.91)**	
	Recessive	AG/GG	42	255	1.00	0.04	1.00	**0.03**
		AA	6	12	3.036 (1.08–8.53)		**3.21 (1.14–9.08)**	

Recurrence/metastasis^a^								
Negative	Codominant	GG	35	161	1.00	0.04	1.00	**0.03**
		AG	36	94	1.76 (1.04–2.99)		**1.82 (1.06–3.10**)	
		AA	7	12	2.68 (0.99–7.30)		**2.84 (1.03–7.80)**	
	Dominant	GG	35	161	1.00	0.02	1.00	**0.01**
		AA/AG	43	106	1.87 (1.12–3.105)		**1.93 (1.16–3.23**)	
	Recessive	AG/GG	71	255	1.00	0.13	1.00	0.12
		AA	7	12	2.095 (0.795–5.52)		2.18 (0.82–5.80)	

Positive	Codominant	GG	41	161	1.00	0.49	1.00	0.58
		AG	29	94	1.21 (0.71–2.08)		1.17 (0.68–2.03)	
		AA	4	12	1.31 (0.40–4.27)		1.41 (0.43–4.63)	
	Dominant	GG	41	161	1.00	0.45	1.00	0.51
		AA/AG	33	106	1.22 (0.3–2.06)		1.20 (0.70–2.03)	
	Recessive	AG/GG	70	255	1.00	0.74	1.00	0.64
		AA	4	12	1.21 (0.38–3.88)		1.32 (0.41–4.27)	

^1^Adjusted by age. *∗*Stage 0 was added to the clinical stage I/II. ^a^Patient numbers may not add up to 100% of available subjects because of missing clinical data.

## Data Availability

The data used to support the findings of this study are available from the authors upon reasonable request and with permission from the Air Force Military Medical University (Xi'an, China).

## Supplementary Materials

Supplementary Materials The supplementary materials include the following: Supplementary Table 1: basic information of candidate SNP in all individuals in this study. Supplementary Table 2: genotype distributions between male and females. Supplementary Table 3: characteristics and clinical features of the gastric cancer group and the control group. Supplementary Table 4: characteristics and clinical features of the female gastric cancer group and the control group. Supplementary Table 5: stratification analyses between GJA1 rs2071165 polymorphism and gastric cancer prognosis.
