# Supplementary material for: GJA1 rs2071165 A > G Variant Increased Gastric Cancer Risk in Females of Northwest China: A Case-Control Study
Source: J Oncol. 2021 May 19;2021:5556303. doi: 10.1155/2021/5556303 (PMC8225425; doi:10.1155/2021/5556303)
Supplement: Supplementary Materials — The supplementary materials include the following: Supplementary Table 1: basic information of candidate SNP in all individuals in this study. Supplementary Table 2: genotype distributions between male and females. Supplementary Table 3: characteristics and clinical features of the gastric cancer group and the control group. Supplementary Table 4: characteristics and clinical features of the female gastric cancer group and the control group. Supplementary Table 5: stratification analyses between GJA1 rs2071165 polymorphism and gastric cancer prognosis. [file 5556303.f1.docx]

Supplementary Table 1 Basic information of candidate SNP in all individuals in this study

| Gene | SNP | Type | Chromosome | Position | Band | Alleles A/B | MAF | |
| --- | --- | --- | --- | --- | --- | --- | --- | --- |
|  |  |  |  |  |  |  | Case | Control |
| GJA1 | rs2071165 | upstream-variant-2KB | 6 | 121435063 | 6q22.31 | A/G | 0.249 | 0.250 |

Supplementary Table 2 Genotype distributions between male and females

|  | | rs2071165 | | | *p* |
| --- | --- | --- | --- | --- | --- |
|  |  | AA | AG | GG |  |
| control | Male | 33(6.7%) | 194(39.7%) | 262(53.6%) | 0.152 |
|  | Female | 12(4.5%) | 94(35.2%) | 161(60.3%) |  |
| case | Male | 30(5.7%) | 191(36.3%) | 305(58.0%) | 0.211 |
|  | Female | 11(7.1%) | 66(42.9%) | 77(50%) |  |

Supplementary Table 3 Characteristics and clinical features of the gastric cancer group and the control group.

| Variables | Gastric Cancers  N (%) | Controls  N (%) | *p* |
| --- | --- | --- | --- |
| **Age (years),( mean±SD)** | 57.57 ±10.826 | 52.58±8.71 | **<0.001** |
| <55 | 251(37) | 429(57) |  |
| >55 | 430(63) | 327(43) |  |
| **Gender(%)** |  |  |  |
| Male, n(%) | 527(77 | 489 (65) | **<0.001** |
| Female, n(%) | 154 (23) | 267 (35) |  |
| **Tumor Clinical stage** |  |  |  |
| 0/I/II | 458(69) |  |  |
| III/ IV | 204(31) |  |  |
| **Tumor diameter(cm)** |  |  |  |
| <5 | 380(57) |  |  |
| ≥5 | 273(41) |  |  |
| **Recurrence/ metastasis** |  |  |  |
| negative | 377(57) |  |  |
| positive | 287(43) |  |  |
| **Position** |  |  |  |
| Cardia | 125(19) |  |  |
| No-cardia | 425(64) |  |  |

Supplementary Table 4. Characteristics and clinical features of in Females gastric cancer group and the control group

| Variables | Gastric Cancers  N (%) | Controls  N (%) |
| --- | --- | --- |
| **Age (years)** |  |  |
| <55 | 77(50) | 87(33) |
| ≥55 | 77(50) | 180(67) |
| **Tumor Clinical stage** |  |  |
| 0/I/II | 104(68) |  |
| III/ IV | 48(31) |  |
| **Tumor diameter(cm)** |  |  |
| <5 | 80(52) |  |
| ≥5 | 69(45) |  |
| **Recurrence/ metastasis** |  |  |
| negative | 78(51) |  |
| positive | 74(48) |  |

Supplementary Table 5. Stratification analyses between *GJA1* rs2071165 polymorphism and gastric cancer prognosis

|  |  | Genotype | OS | | | | RFS | | | |
| --- | --- | --- | --- | --- | --- | --- | --- | --- | --- | --- |
|  |  |  | Total | Event | HR (95%CI) | *p* | Total | Event | HR (95%CI) | *p* |
| age | <60 | GG | 206 | 62 |  | 0.528 | 206 | 86 |  | 0.175 |
|  |  | AG | 144 | 50 | 1.092(0.698-1.709) | 0.701 | 144 | 62 | 0.845(0.57-1.252) | 0.401 |
|  |  | AA | 25 | 10 | 1.535(0.729-3.231) | 0.259 | 25 | 11 | 1.641(0.828-3.253) | 0.156 |
|  | ≥60 | GG | 169 | 63 |  | 0.789 | 169 | 81 |  | 0.666 |
|  |  | AG | 106 | 35 | 0.881(0.554-1.401) | 0.593 | 106 | 40 | 0.84(0.547-1.29) | 0.426 |
|  |  | AA | 13 | 5 | 0.728(0.213-2.49) | 0.613 | 13 | 7 | 1.149(0.439-3.006) | 0.777 |
| sex | female | GG | 76 | 33 |  | 0.316 | 76 | 41 |  | 0.6 |
|  |  | AG | 65 | 22 | 0.671(0.343-1.312) | 0.244 | 65 | 29 | 0.739(0.41-1.331) | 0.314 |
|  |  | AA | 11 | 3 | 0.419(0.112-1.568) | 0.196 | 11 | 4 | 0.828(0.266-2.582) | 0.745 |
|  | male | GG | 299 | 92 |  | 0.486 | 299 | 126 |  | 0.274 |
|  |  | AG | 185 | 63 | 1.21(0.838-1.746) | 0.31 | 185 | 73 | 0.932(0.669-1.299) | 0.678 |
|  |  | AA | 27 | 12 | 1.36(0.67-2.762) | 0.395 | 27 | 14 | 1.598(0.846-3.018) | 0.149 |
| HP | no | GG | 110 | 30 |  | 0.665 | 110 | 48 |  | 0.374 |
|  |  | AG | 68 | 21 | 1.087(0.597-1.977) | 0.785 | 68 | 29 | 0.939(0.574-1.538) | 0.804 |
|  |  | AA | 8 | 3 | 1.755(0.514-5.987) | 0.369 | 8 | 4 | 2.035(0.701-5.905) | 0.191 |
|  | Yes | GG | 221 | 71 |  | 0.811 | 221 | 87 |  | 0.54 |
|  |  | AG | 147 | 46 | 0.977(0.666-1.432) | 0.905 | 147 | 51 | 0.848(0.595-1.208) | 0.362 |
|  |  | AA | 24 | 9 | 0.787(0.38-1.627) | 0.518 | 24 | 11 | 1.149(0.598-2.208) | 0.677 |
| BMI | BMI≥24 | GG | 88 | 28 |  | 0.867 | 88 | 37 |  | 0.69 |
|  |  | AG | 67 | 16 | 1.104(0.768-1.585) | 0.594 | 67 | 19 | 0.95(0.686-1.314) | 0.755 |
|  |  | AA | 8 | 2 | 1.032(0.523-2.036) | 0.928 | 8 | 4 | 1.265(0.668-2.399) | 0.471 |
|  | BMI<24 | GG | 243 | 73 |  | 0.763 | 243 | 98 |  | 0.193 |
|  |  | AG | 148 | 51 | 0.787(0.414-1.494) | 0.463 | 148 | 61 | 0.665(0.367-1.203) | 0.177 |
|  |  | AA | 24 | 10 | 0.966(0.221-4.219) | 0.964 | 24 | 11 | 1.7(0.577-5.004) | 0.336 |
| tumor size |  | GG | 222 | 51 |  | 0.93 | 222 | 77 |  | 0.704 |
|  | < 5 cm | AG | 140 | 39 | 1.077(0.671-1.729) | 0.758 | 140 | 47 | 0.921(0.611-1.387) | 0.692 |
|  |  | AA | 14 | 2 | 0.875(0.211-3.63) | 0.854 | 14 | 4 | 1.427(0.515-3.957) | 0.494 |
|  |  | GG | 145 | 71 |  | 0.997 | 145 | 87 |  | 0.448 |
|  | ≥5 cm | AG | 106 | 45 | 1.013(0.654-1.569) | 0.953 | 106 | 53 | 0.848(0.566-1.272) | 0.425 |
|  |  | AA | 22 | 12 | 0.988(0.484-2.016) | 0.973 | 22 | 13 | 1.295(0.655-2.562) | 0.457 |
| clinical stage | early | CC | 71 | 6 |  | 0.585 | 71 | 9 |  | 0.682 |
|  |  | TC | 61 | 4 | 0.572(0.103-3.186) | 0.524 | 61 | 5 | 0.805(0.212-3.065) | 0.751 |
|  |  | TT | 8 | 1 | 2.073(0.206-20.852) | 0.536 | 8 | 1 | 2.237(0.246-20.332) | 0.475 |
|  | middle | CC | 270 | 94 |  | 0.784 | 270 | 128 |  | 0.465 |
|  |  | TC | 168 | 67 | 1.125(0.783-1.617) | 0.523 | 168 | 83 | 1.04(0.754-1.435) | 0.809 |
|  |  | TT | 25 | 10 | 1.165(0.557-2.436) | 0.686 | 25 | 13 | 1.489(0.793-2.797) | 0.216 |
| ACT | late | CC | 32 | 25 |  | 0.718 | 32 | 29 |  | 0.177 |
|  |  | TC | 20 | 13 | 1.375(0.598-3.165) | 0.454 | 20 | 13 | 0.693(0.324-1.479) | 0.343 |
|  |  | TT | 3 | 3 | 1.408(0.371-5.35) | 0.615 | 3 | 3 | 2.467(0.653-9.312) | 0.183 |
|  | negative | CC | 135 | 31 |  | 0.637 | 135 | 34 |  | 0.264 |
|  |  | TC | 83 | 23 | 1.272(0.705-2.296) | 0.424 | 83 | 25 | 1.349(0.766-2.378) | 0.3 |
|  |  | TT | 14 | 3 | 1.523(0.443-5.234) | 0.504 | 14 | 4 | 2.252(0.762-6.659) | 0.142 |
|  | positive | CC | 240 | 94 |  | 0.929 | 240 | 133 |  | 0.12 |
|  |  | TC | 167 | 62 | 0.928(0.633-1.361) | 0.702 | 167 | 77 | 0.74(0.529-1.034) | 0.077 |
|  |  | TT | 23 | 12 | 0.973(0.469-2.02) | 0.942 | 23 | 14 | 1.244(0.649-2.386) | 0.511 |
